# Ophthalmology

**Published:** 1891-09

**Authors:** Alvin A. Hubbell


### OPHTHALMOLOGY.

By ALVIN A. HUBBELL, M. D.

### INFLUENZA AS A CAUSE OF OPTIC NEURITIS.

Macnamara, of London, in a recent address, (*British Medical Journal*, August 1, 1891,) alludes to neuritis of the optic nerve occurring in conjunction with influenza, and as appearing analogous to cases which he has described as "malarial peripheral neuritis." He refers to four cases, three male patients and one female, suffering from double optic neuritis, which came on while they were passing through an attack of influenza. In these cases the patients' sight had been perfectly good until they were seized with influenza, and there was no evidence of any intracranial affection, albuminuria, or any other cause with which could be associated the affection of the optic papilla from which they suffered. In the first case that came under observation, the patient had been absolutely blind for six weeks before he saw him. There was intense papillitis with numerous retinal hemorrhages. Both optic discs became atrophied and the patients did not regain the slightest perception of light. The other cases were recent and showed uncomplicated papillitis with great impairment of vision. The discs in all three cases gradually cleared up, mercurial treatment being used, and the patients regained perfect vision. He suggests that the neuritis depends "upon either direct local irritation produced by microbes on the optic nerve, or on the ptomaines secreted by these micro-organisms."

---

Dr. Weeks, of New York, (*New York Medical Journal*, August 8, 1891,) has also written an interesting paper on forms of optic neuritis, caused by *la grippe* or influenza. He reviews cases published by others, and cites one case of his own. In all, he collects fifteen cases. Of these, "nine occurred in females and six in males, at ages ranging from eighteen to fifty-eight years. There was an inflammatory condition of the disc in four cases; paling of the disc, more or less marked, in eleven cases; one eye was affected in four cases, both eyes in eleven. Blindness (permanent) resulted in one eye in one case; perception of light in two eyes in two cases. Approximately, complete recovery occurred in one case only. The scotomata produced were very varied, affecting all parts of the visual fields. Central scotoma without appreciable limitation of the visual fields occurred in two cases. The scotomata were for *all colors* except in one case." Weeks draws the following conclusions:

1. Neuritis of the optic nerve due to *la grippe* is of relatively rare occurrence; it may affect one or both eyes, and may produce partial transient impairment of vision, partial permanent impairment of vision, or absolute permanent blindness.

2. Failure of vision begins from three to fourteen days after the commencement of the attack of *la grippe*, and proceeds quite rapidly. It is always preceded by intense frontal or circumorbital cephalalgia.

3. The form of scotoma produced is probably dependent on the position of the neuritis in the course of the nerve from the globe to the chiasm. If immediately behind the globe, the macular fibres are affected; if near the optic foramen, the peripheral fibres suffer first.

4. Treatment has but little effect to promote cure. If recovery follows, it takes place spontaneously and accompanies improvement in the patients' general condition.

